# Optical Behavior of Human Skin Substitutes: Absorbance in the 200–400 nm UV Range

**DOI:** 10.3390/biomedicines10071640

**Published:** 2022-07-08

**Authors:** Javier Ruiz-López, Juan C. Cardona, Ingrid Garzón, María M. Pérez, Miguel Alaminos, Jesus Chato-Astrain, Ana M. Ionescu

**Affiliations:** 1 Department of Optics (Laboratory of Biomaterials Optics), Faculty of Science, University of Granada, E18071 Granada, Spain; jruizlo@ugr.es (J.R.-L.); cardona@ugr.es (J.C.C.); mmperez@ugr.es (M.M.P.); anaionescu@ugr.es (A.M.I.); 2 Instituto de Investigación Biosanitaria ibs.GRANADA, E18011 Granada, Spain; igarzon@ugr.es (I.G.); malaminos@ugr.es (M.A.); 3 Department of Histology (Tissue Engineering Group), Faculty of Medicine, University of Granada, E18016 Granada, Spain

**Keywords:** absorption, UV radiation, bioengineered skin, fibrin-agarose biomaterial

## Abstract

The most recent generation of bioengineered human skin allows for the efficient treatment of patients with severe skin defects. Despite UV sunlight can seriously affect human skin, the optical behavior in the UV range of skin models is still unexplored. In the present study, absorbance and transmittance of the UGRSKIN bioartificial skin substitute generated with human skin cells combined with fibrin-agarose biomaterials were evaluated for: UV-C (200–280 nm), -B (280–315 nm), and -A (315–400 nm) spectral range after 7, 14, 21 and 28 days of ex vivo development. The epidermis of the bioartificial skin substitute was able to mature and differentiate in a time-dependent manner, expressing relevant molecules able to absorb most of the incoming UV radiation. Absorbance spectral behavior of the skin substitutes showed similar patterns to control native skin (VAF > 99.4%), with values 0.85–0.90 times lower than control values at 7 and 14- days and 1.05–1.10 times the control values at 21- and 28-days. UV absorbance increased, and UV transmission decreased with culture time, and comparable results to the control were found at 21 and 28 days. These findings support the use of samples corresponding to 21 or 28 days of development for clinical purposes due to their higher histological similarities with native skin, but also because of their absorbance of UV radiation.

## 1. Introduction

The human skin is the largest and outermost organ of the human body and serves as the external protective barrier between the organism and the environment. The skin is composed of the epidermis, dermis and hypodermis, and they are functionally orchestrated to fulfill a vast role in protection and resistance to environmental agents such as microorganisms, chemical agents and ultraviolet light (UV) [1]. This protective function is mainly provided by the epidermal layer, which forms a tight barrier that is essential to prevent pathogen invasion, and to stop chemical agents from accessing the inner tissues and regulate water loss. In addition, the epidermal barrier plays a major role in protecting the cells and tissues from damage caused by incoming light. In fact, sunlight exposure may have severe biological and clinical consequences on the skin, ranging from acute sunburn reactions and tanning to long-term effects ranging from hyperpigmentation to photoaging and photocancer [2,3,4]; most of these effects are caused by UV.

In general, sunlight consists of a mixture of visible and non-visible light, with UV being among the most energetic wavelengths found in this radiation. Sunlight UV can be classified as UV-C (200–280 nm), UV-B (290–320 nm) and UV-A radiations (320–400 nm) [3,5] which can seriously affect the human skin dermis and epidermis. The potentially harmful UV-C radiation is normally absorbed by the ozone atmosphere. However, UV-B and UV-A are able to reach the biosphere and can interact with the human skin. UV-B can directly induce DNA lesions such as cyclobutene pyrimidine dimers or 6, 4 photoproducts [6]. UV-A radiation, on the contrary, is less energetic, but has higher penetration properties and can reach the epidermal and dermal layers and generate reactive oxygen species (ROS) [7]. Protection against these effects of UV is one of the major functions of the skin barrier, and strictly depends on the presence of different types of intercellular junctions and proteins associated with this barrier [8].

As the outermost organ, the skin is subjected to multiple diseases and conditions, with severe burns being a significant cause of pathology able to disrupt its barrier function. Although localized lesions can be treated with autografts, severe cases are very difficult to manage due to the lack of healthy donor areas and the limitations of this procedure [8,9]. Therefore, it is necessary to develop alternative approaches for the clinical treatment of these patients.

In this context, tissue engineering approaches allow the generation of human skin substitutes in the laboratory which can be used in clinical applications [10]. In recent years, different skin substitutes have been engineered worldwide [11,12]. Although most skin substitutes have been described for laboratory use and are still in a preclinical stage, some substitutes were able to fulfill all the requirements for clinical use and have been used clinically for the treatment of skin defects [13]. Numerous skin substitutes have been developed at the preclinical level, with most of these models based on collagen, chitosan, gelatin, alginate or other similar biomaterials [14]. In addition, very few skin substitutes have been clinically evaluated in Europe as Advanced Therapy Medicinal Products (ATMP), including a few commercial skin analogues [13,15]. However, the clinical usefulness of most skin substitutes is still under validation [16,17,18,19].

Among the different skin substitutes showing results, a model of bioengineered human skin originally generated at the University of Granada [20] called UGRSKIN demonstrated to be potentially useful at the preclinical level [21,22,23]. UGRSKIN consists of a fibrin-agarose dermal substitute containing human fibroblasts and a stratified epithelium on top generated with autologous cultured keratinocytes. This skin model was approved by the Spanish Medicines Agency for use in severely burnt patients as an ATMP in a specific hospital in Spain using the hospital exception rule for this type of medicinal product [24]. The preliminary results in patients with large skin burns treated in the Andalusian Public Healthcare System were promising, although the UGRSKIN model will still need to be validated in larger cohorts of patients [25]. Despite the positive preliminary results, further research is needed in order to better understand the properties of these bioartificial tissues and thus improve their clinical efficiency. The UGRSKIN model consists of a biocompatible fibrin-agarose biomaterial containing human dermal fibroblasts within the biomaterial, and an epithelial layer on top generated with cultured human keratinocytes [20]. This skin substitute was approved by the Spanish Medicines Agency for autologous clinical use and is currently being applied for the treatment of severely burned patients [25].

A previous biomechanical and optical characterization of the UGRSKIN model showed that this skin substitute shared several physical similarities with human native skin [21]. The analysis of the optical properties of UGRSKIN in the visible light range demonstrated that the transmittance, absorption and scattering parameters were comparable to the native skin, especially after specific periods of development in culture [21]. However, the optical properties of the UGRSKIN model were not investigated in the UV range. As this is a very important parameter of bioartificial skin substitutes, in the present work we have evaluated the optical behavior of these substitutes in the UV-A, UV-B and UV-C ranges. Results will contribute to the better characterization of this skin substitute and could allow further optimization for future clinical use. The aim of this study is to determine the behavior of the UGRSKIN model to contribute to its preclinical characterization as an ATMP for use in severely burned patients. In addition, our objective was to demonstrate the usefulness of this model as a protective barrier from the incoming sunlight, and to determine the development stage when the artificial tissue is able to protect the inner tissues from the harmful UV light.

## 2. Materials and Methods

### 2.1. Tissue Samples and Cell Isolation

Primary cell cultures of human skin keratinocytes and fibroblasts were established from small full-thickness skin biopsies obtained from healthy donors who underwent skin surgery. Immediately after excision, skin samples were kept at 4 °C in Dulbecco’s Modified Eagle’s Medium (DMEM; Merck, Darmstadt, Germany), supplemented with antibiotics and antimycotics (100 U/mL penicillin G, 100 mg/mL streptomycin, and 0.25 mg/mL amphotericin B; Merck, Darmstadt, Germany), and delivered to the laboratory. Biopsy samples were then washed twice in phosphate-buffered saline (PBS), surgically prepared to remove any remaining adipose tissue, and processed to establish primary cell cultures of keratinocytes and fibroblasts. To obtain human skin keratinocyte cultures, biopsies were carefully rinsed in 1× PBS and the explant technique was used to obtain small fragments of epidermal tissue as previously described [26]. Tissue fragments were seeded into a culture flask and incubated overnight at 37 °C with 1 mL of keratinocyte growth medium to prevent cells from detaching. Subsequently, 1 mL of medium was added every day until a final volume of 5 mL was reached. The keratinocyte growth medium consisted of a 3:1 mixture of DMEM and Ham’s F12 supplemented with 10% fetal calf serum, 100 U/mL penicillin, 0.1 mg/mL streptomycin, and 0.25 g/mL amphotericin B, 24 µg/mL adenine, 0.4 mg/mL hydrocortisone, 5 mg/mL insulin, 10 ng/mL epidermal growth factor, and 1.3 ng/mL triiodothyronine (Merck, Darmstadt, Germany). To obtain skin fibroblast cultures, dermal fragments were enzymatically digested using 2 mg/mL Clostridium histolyticum collagenase I (Gibco-BRL, Thermo Fisher Scientific, Waltham, MA, USA) at 37 °C for 6 h. Isolated fibroblasts were collected by centrifugation and expanded in culture flasks containing basal cell culture medium (DMEM supplemented with 10% fetal bovine serum, 100 U/mL penicillin, 0.1 mg/mL streptomycin, and 0.25 g/mL amphotericin B, all from Merck) under standard cell culture conditions.

### 2.2. Generation of Human Skin Substitutes by Tissue Engineering

Once primary cell cultures of human skin keratinocytes and fibroblasts were obtained, human skin substitutes were developed using fibrin-agarose based biomaterials as previously described [20,21]. In brief, a bioartificial dermal skin layer substitute was first generated by mixing 760 µL of human plasma as a fibrin source, 75 μL of DMEM containing 140,000 cultured human fibroblasts, 15 µL of tranexamic acid—as an antifibrinolytic agent—(Amchafibrin, Fides-Ecofarma, Valencia, Spain), 50 μL of a 2% solution of type VII agarose (Merck, Darmstadt, Germany) melted in PBS, and 100 μL of 1% CaCl2 solution (Merck, Darmstadt, Germany) per ml of mixture. This mixture was rapidly aliquoted in Transwell cell culture inserts with 0.4 μm porous membranes and 24 mm of diameter (Corning-Costar, Corning, NY, USA) and allowed to jellify at 37 °C for at least six h. Once jellified, an epidermal layer was generated by subculturing human keratinocytes, which were placed on top of the dermal substitute. Stratification and differentiation of the epithelial layer were promoted using the air-liquid culture technique. This technique was performed by reducing the amount of culture medium in the porous inserts so that only the stromal substitute remained submerged, whereas the developing epithelium was in direct contact with air and received nutrition from the stromal layer [20,21]. The bioengineered skin was kept for 7, 14, 21 and 28 days in a cell incubator, using standard cell culture conditions (37 °C and 5% CO_2_). After each follow-up period, human skin substitutes were subjected to plastic compression nanostructuration techniques as previously described [27].

### 2.3. Histological Analysis

Human skin substitutes and native human skin control samples were fixed in 4% formaldehyde and embedded in paraffin to obtain 4 μm-thick histological sections. Tissue sections were dewaxed, rehydrated, and stained for histological analysis. To evaluate tissue morphology and structure, sections were stained with hematoxylin-eosin (HE) (Panreac AppliChem, Barcelona, Spain). Nuclear detection was performed with 4′,6-diamidino-2-fenilindol (DAPI) (Vector laboratories, Burlingame, CA, USA). To evaluate epidermis development and differentiation, tissue sections were subjected to immunohistochemical analysis for pancytokeratin (PANCK, a broad-spectrum marker of epithelial tissue), cytokeratin 10 (CK10, a marker of epithelial stratification and maturation), cytokeratin 5 (CK5, a marker of epithelial cell proliferation) and filaggrin (FLG, a marker of epithelial differentiation and skin barrier function). In brief, tissue sections were deparaffinized and subjected to antigen retrieval with pH 8 EDTA buffer (25 min at 95 °C) for PANCK, or with pH 6 citrate buffer (25 min at 95 °C) for CK10, CK5 and FLG, and endogenous peroxidase was quenched with H_2_O_2_. Samples were then preincubated in a blocking solution containing horse serum and incubated with one of the following primary antibodies: anti-PANCK (MAD-000755QD), anti-CK10 (MAD-000150QD), anti-CK5 (MAD-210651Q) (all from Master Diagnostica, Granada, Spain, prediluted) and anti-FLG (ab221155, Abcam, Cambridge, UK, dilution 1:5000). After washing in PBS, tissues were incubated in secondary anti-mouse or anti-rabbit antibodies labelled with peroxidase (ImmPRESS reagent kit, Vector Laboratories; prediluted), washed in PBS, and incubated with diaminobenzidine (DAB) (Vector laboratories, Newark, CA, USA). In all cases, positive and negative control tissues were used, and samples were counterstained using Harry’s hematoxylin and coverslipped. To histologically evaluate the dermis development, relevant extracellular matrix components (ECM) were assessed by identifying collagen fibers and proteoglycans using alcian blue (AB) and picrosirius red (PS) histochemistry, respectively, as previously reported [28] (reagents from Panreac AppliChem, Barcelona, Spain). Normal human native skin samples (CTR) were used as controls for all these analyses.

### 2.4. Optical Properties

The absorbance (A) and transmittance (T) of UV radiation propagating throughout the human skin substitutes in the 200–400 nm spectral range were calculated from the intensities measured using a spectrometer (Thorlabs CCS200/M, 200–1000 nm) with a spectral light source (BDS130 Deuterium/Tungsten, 190–2500 nm) as:

(1) $$A=\log\frac{I_{0}}{I}=\log\frac{1}{T}$$

where *I*_0_ is the incident light intensity and *I* is the transmitted light intensity. Its ratio determines the inverse of light transmission *T* (Equation (1)).

Human skin substitutes were measured after 7, 14, 21 and 28 days of development in culture. Native human skin samples considered as controls (CTR) underwent the same measurement protocol. The thickness of all samples was measured using a Nikon Eclipse 90i light microscope.

The normalized absorbance (An) was also calculated, with normalization being performed with respect to the control samples measurements. Optical properties were analyzed for: UV-C (200–280 nm), -B (280–315 nm) and -A (315–400 nm) spectral ranges [29].

### 2.5. Quantification of Histological Parameters and Statistical Analysis

To determine the histological development level of the epidermis upon the culture time, we first quantified the thickness of the epidermis and the number of epithelial cell strata found at day 7, 14, 21 and 28 of development. For the epidermal thickness, we used the line selection tool of ImageJ software (National Institutes of Health, Bethesda, MD, USA) and measured the height of the epithelium at 20 independent points in each image, using the scale bar as a control. For the number of layers, we quantified the number of cell strata found at 20 independent points of the epidermal layer. We then analyzed the presence of the ECM components, the collagen and the proteoglycans. With this purpose, we used the square tool ImageJ (25 µm × 25 µm squares) and quantified the average pixel intensity within each square, as previously described [30,31,32,33]. In each image, 20 independent areas were randomly selected at the dermal substitute. For the immunohistochemical analysis, expression was semiquantitatively assessed as negative (0), slightly positive (1), positive (2), or strongly positive (3), as previously reported [34].

For the statistical analysis of the histological and optical parameters, we first analyzed the normality of the distributions using the Shapiro-Wilk test and the homogeneity of variances using a Levene’s test. As both tests demonstrated that the criteria required for parametrical testing was not fulfilled, we used non-parametric statistical analysis. To globally compare the results corresponding to several groups at the same time (for example, A vs. An vs. T), we used the Kruskal-Wallis one-way analysis of variance by ranks. For pair-wise comparisons between two specific groups, we used the Mann-Whitney U test, and the tau test of Kendall was used in correlation analyses. Comparisons were carried out for each development time and for each UV range. For the optical parameters, the variance accounted for value (VAF) was also calculated for each UV range as a determination of the variation of the trend of spectral distribution of A, An and T during time in culture, compared with the control sample.

$$VAF\;\left(\%\right)=\frac{{\left(\sum_{k=i}^{j}a_{k}b_{k}\right)}^{2}}{\left(\sum_{k=i}^{j}a_{k}^{2}\right)\left(\sum_{k=i}^{j}b_{k}^{2}\right)}$$

where $a_{k}$ is the value of the parameter to be studied (for each wavelength) and $b_{k}$ is the equivalent for another measurement.

A Bonferroni-corrected *p* value of 0.001 was set, as multiple testing was performed. The statistical analysis was performed using a standard statistical software package (SPSS Statistics 20.0.0, IBM, Armonk, NY, USA).

## 3. Results

### 3.1. Histological Characterization of Human Skin Substitutes

**Figure 1:**
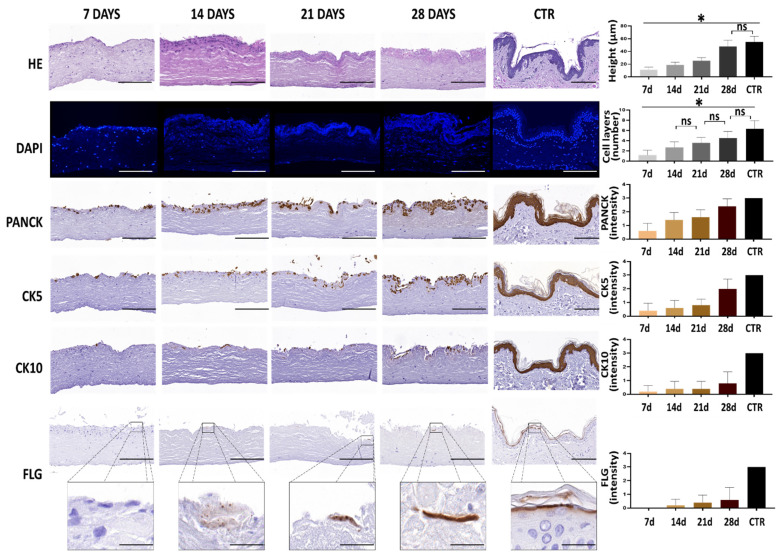
Histological evaluation of the epidermis of the human skin substitutes and native human skin control. The histological features of the epidermis human skin substitutes developed after 7, 14, 21 and 28 days and human controls (CTR) were evaluated with Hematoxylin-Eosin (HE) and DAPI, as well as with key epithelial markers using immunohistochemistry techniques for PANCK, CK5, CK10 and FLG. The thickness of the epidermis, the number of epithelial cell strata and the immunohistochemical positive expression were quantified and shown accordingly. Statistically significant differences between groups are labeled with asterisks (*), whereas non-significant differences are labeled with “ns”. Scale bar = 200 µm and 26 µm in the insets.

We performed a histological analysis of the bioengineered human skin substitutes kept in culture for 7, 14, 21 and 28 days. This analysis revealed the presence of a developing epidermis on top of the fibrin-agarose dermal substitute that differed among the study times (Figure 1). At day seven of follow-up, this epithelium was thin and consisted of a single cell layer. However, the thickness and the number of layers of the epithelium significantly increased over time, with a significant correlation between thickness and time (*p* < 0.0001, r = 0.8049) and between cell number and time (*p* < 0.0001, r = 0.6510). Global comparisons using the Kruskal-Wallis test revealed significant differences among groups (*p* < 0.0001). When specific groups were compared using Mann-Whitney tests, we found significant differences (*p* < 0.0001) between CTR samples and bioartificial tissues corresponding to 7, 14 and 21 days of development, but not between CTR and 28-days bioartificial tissues, which resulted in non-significance (*p* = 0.0227 for the epithelial thickness and *p* = 0.0016 for the number of cell strata). Pair-wise comparisons between two specific development times were always significant, except for the comparison of day 14 vs. day 21 and day 21 vs. day 28 for the number of cell layers (Figure 1).

In addition, we analyzed several key epithelial markers using immunohistochemistry. As shown in Figure 1, our expression analysis first showed the intense expression of PANCK and CK 5 and 10 in the CTR skin epidermis. We then found that bioengineered human skin expressed increasing amounts of these markers, with a significant correlation with the culture time (*p* = 0.0003 and r = 0.7106 for PANCK, *p* = 0.0016 and r = 0.6231 for CK5 and *p* = 0.0368 and r = 0.4224 for CK10). In general, the bioartificial skin kept in culture for seven days showed low amounts of PANCK, CK5 and CK10. However, the expression of these three markers tended to increase at days 14 and 21 and, especially, at day 28 of ex vivo culture, although the expression levels found in CTR skin were not reached. In general, the expression of PANCK tended to be higher than CK5, with the lowest expression corresponding to CK10. For CK10, expression was found in scattered epithelial cells, suggesting that specific cells could be committed to differentiation and maturation. For the epithelial differentiation marker filaggrin, our results showed that the bioartificial skin samples kept in culture expressed low amounts of this marker (slight expression), with a moderate increase over time that did not reach the expression found in CTR skin (Figure 1).

**Figure 2:**
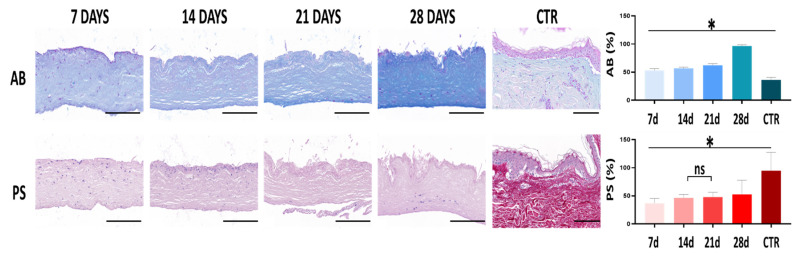
Histological evaluation of the dermis of the human skin substitutes and native human skin control. The histological features of the dermis human skin substitutes developed after 7, 14, 21 and 28 days and human controls (CTR) were assessed using Alcian Blue (AB) and Picrosirius (PS) for extracellular matrix components (ECM) evaluation, proteoglycans, and collagen fibers, respectively. Proteoglycans and collagen fibers expression were quantified in AB and PS-stained sections; values correspond to reaction intensity for each histochemical method. Statistically significant differences between groups are labeled with asterisks (*), whereas non-significant differences are labeled with “ns”. Scale bar = 200 µm.

In the second place, we analyzed the histological structure of the dermal layer of the bioartificial skin samples and controls. In this regard, we found that the bioartificial human skin consisted of a dense fibrillar biomaterial containing abundant fibroblasts immersed within this biomaterial. Although this structure was partially analogue to the extracellular matrix found in CTR samples, blood vessels and the typical organization in papillary and reticular dermis of the CTR were not present in the bioartificial samples. In fact, bioengineered human skin did not show the typical rete-ridges and papillae skin specializations, and the dermo-epithelial junction was rather flat (Figure 2).

When the composition of the dermal layer was analyzed using AB and PS histochemistry (Figure 2), we found significant differences associated to the sample type and development time. On the one hand, the expression analysis of proteoglycans as determined by AB revealed that bioengineered samples were significantly enriched in these non-fibrillar components of the extracellular matrix compared to the CTR dermis (*p* < 0.0001). In addition, the presence of proteoglycans tended to increase with culture time in bioengineered skin samples, with a significant correlation with time (*p* < 0.0001 and r = 0.7980). Differences between specific culture times were always statistically significant (*p* < 0.0001). For the fibrillar components, our analysis showed that CTR samples contained significantly higher amounts of fibers than the bioengineered skin (*p* < 0.0001), although the fibers content tended to significantly increase with culture time (*p* = 0.0001 and r = 0.2168) without reaching CTR levels. Differences between times were significant (*p* < 0.0001) except for the comparison of day 14 vs. day 21 in bioartificial samples (*p* = 0.7600).

### 3.2. Optical Characterization of the Human Skin Substitutes

**Figure 3:**
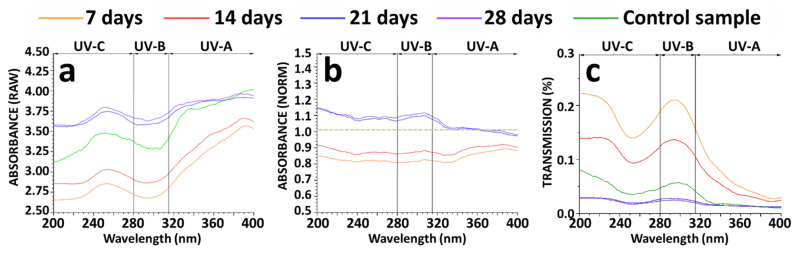
Absorbance and transmittance optical properties of the human skin substitutes and native human skin control (CTR). (**a**,**b**) The absorbance, the normalized absorbance and the transmission were studied during 7, 14, 21 and 28 days of in vitro development in culture. (**c**) Native human skin was used as the control sample and for the absorbance normalization. The transmission was calculated as a function of wavelength.

As shown in Figure 3, we analyzed the optical properties of the spectral behavior of CTR and bioartificial skin in the 200–400 nm UV range (absorbance, normalized absorbance and transmission) (Figure 3). Results showed that the absorbance spectral behavior (Figure 3a) of the skin substitutes showed similar patterns to CTR skin at all development times, with VAF values higher than 99.47% for all UV ranges. However, we found that the absorbance values were significantly lower in seven- and 14-days bioengineered skin samples than in CTR skin (*p* < 0.0001), whereas 21-days and 28-days samples were significantly higher than CTR in the UV-C and UV-B ranges (*p* < 0.0001). In contrast, in the UV-A range, starting with approximately 370 nm, the CTR skin displayed slightly higher absorbance values with statistically significant differences with the 21 and 28-days of development in culture samples (*p* < 0.0001). Nevertheless, bioengineered skin samples corresponding to 28 days of development did not differ from 21-days samples for the UV-C and UV-A ranges (*p* = 0.0039 and *p* = 0.0028, respectively). These results were confirmed when the absorbance values of the bioengineered skin samples were normalized to CTR samples (Figure 3). After normalization, the spectral behavior of all bioengineered skin samples was similar in all UV ranges at all periods of time (VAF > 99.66%).

In addition, the bioengineered skin corresponding to seven and 14 days of development displayed absorbance values 0.85–0.90 times lower than CTR, whereas 21- and 28-days samples showed 1.05–1.10 times the CTR values. In fact, the analysis of the normalized absorbance (Figure 3b) revealed statistically significant differences between samples kept in culture for seven and 14 days and the 21- and 28-days samples (*p* < 0.0001). Moreover, no statistically significant differences were found between 21- and 28-days samples for all UV ranges studied (*p* = 0.0325, *p* = 0.0020 and *p* = 0.0064 for UV-C, B and A, respectively).

These results correlated with the values obtained for the UV light transmission (Figure 3c). In short, our analysis showed that the highest transmission corresponded to bioartificial skin kept in culture for seven and 14 days, whose values were significantly higher than CTR values (*p* < 0.0001), although the spectral behavior of these samples showed similar patterns with the control skin. (VAF > 96.08% in the UV-C, >98.95% in the UV-B and >94.80% in the UV-A ranges). In contrast, transmission corresponding to 21- and 28-days bioengineered skin samples was significantly lower than CTR in the UV-C and UV-B ranges (*p* < 0.0001). For the UV-A range, no statistically significant differences were found between the 21-day samples and the CTR skin (*p* = 0.0129), and also the 21 and 28-days samples (*p* = 0.0034).

## 4. Discussion

A thorough characterization of novel bioartificial tissues generated in the laboratory is one of the essential requirements of medical agencies for tissues intended for clinical use [25,35]. Although our model of bioartificial skin has already been evaluated and characterized at different levels [20,22,26], the optical behavior of the UGRSKIN model in the UV range was not previously evaluated. Since one of the main functions of grafted skin is protecting the patient’s inner tissues from UV light damage, a proper optical evaluation of this ATMP would significantly contribute to establish the putative therapeutic potential of this novel product.

Among the main skin components playing a role in the absorption of incoming light, melanin, opsins and hemoglobin pigments were shown to have a high capability to absorb visible and UV light [36]. Other components of the normal skin such as cytokeratins and collagen fibers are also able to absorb significant amounts of light [37]. In addition, cells and their structural components: nuclei, organelles and other small particles, are important structures of all human tissues that are able to prevent light from entering to the inner structures by absorbing part of the incoming light [21,38]. Although both the dermal fibroblasts and the epidermal keratinocytes can exert this function, it is thought that keratinocytes play a major role in UV light absorbance due to the presence of cytokeratins, opsins and possibly other molecules that are able to interact with UV radiation in the cytoplasm of these cells [37,39].

In the present study, we demonstrated that the bioengineered skin model generated in the laboratory was capable of absorbing most of the incident UV light, thus preventing this highly energetic radiation to reach deeper tissues. In general, our results showed that the UV optical properties and spectral behavior of the bioengineered human skin substitutes cultured ex vivo tended to mimic those of the native human skin, especially in the UV-A range, at the end of the follow-up period. In fact, UV absorbance increased, and UV transmission decreased with culture time, coinciding with the important changes found at the epidermal and dermal compartments of the skin substitutes, and became comparable to CTR after 21 and 28 days of development. In fact, our findings showed that bioartificial skin was initially histologically immature and tended to gradually differentiate in a time-dependent manner. These results are in agreement with previous reports demonstrating that both the epithelial and the stromal layer of bioartificial skin experience a sequential process of development that correlates with time in culture [22,23]. As in these previous reports, the epidermal layer of the bioengineered skin substitutes initially consisted of a single stratum of cells which were able to proliferate and stratify with time in culture to develop a stratified epithelium whose thickness and number of cell layers did not differ from control skin at day 28. This increment in the number of cells found at the epithelial layer coincided with the similar levels of UV light transmission and absorption found in these samples and suggests that the main factor responsible for this phenomenon could be the epidermal keratinocytes that developed on top of the bioengineering skin and became very abundant at longer follow-up times. Interestingly, our analysis showed that the optical behavior was very similar at 21 and 28 days, despite the fact that the number of cells and the epithelial thickness was lower at day 21. It is likely that the number of cells found at day 21 could be sufficient to achieve the UV absorption properties found in CTR skin.

In addition, the sequential epidermal maturation and differentiation of the bioengineered skin was accompanied by a time-dependent expression of key epithelial markers and dermal components, as expected [22,23]. At the epithelial level, our analysis showed a significant correlation between time and the presence of PANCK, CK5 and CK10 in the bioengineered skin epidermis, and samples corresponding to 21 and, especially, 28 days of development had significant amounts of these epithelial components. Most likely, the presence of these molecules was associated with the high UV absorbance found in these samples, and we may hypothesize that the bioengineered human skin corresponding to 21 and 28 days could be able to prevent UV light from reaching the inner structures, thus protecting these structures from non-specific cellular and tissular damage, as it is the case of the native skin [1,40]. Interestingly, the levels of expression of most of these components did not reach those found in normal, native skin, although this finding does not seem to alter the optical properties of the bioengineered skin in the UV range. These results are in agreement with previous reports suggesting that human bioengineered tissues kept in culture are able to mature and differentiate until some extent, but terminal differentiation will require in vivo grafting [22]. Despite the fact that samples were always kept ex vivo and differentiation was partial, it is noteworthy that expression of PANCK and CK5 was higher than CK10 and FLG. This finding is not surprising, since it is well known that CK10 and FLG, which are usually coexpressed, are markers of highly differentiated, cornified epithelia, whereas PANCK and CK5 are present in all epithelia, especially when epithelial cells are proliferating [41]. Although found at low concentrations, FLG tended to be more abundant at days 21 and 28 of development. Its presence has been associated to UV absorption through the formation of urocanic acid, which is, in turn, among the most important non-melanin keratinocyte protectants [42,43,44,45].

Regarding the dermal compartment of the skin, we also found a correlation with the time in culture in bioengineered skin substitutes, which confirms previous studies demonstrating that the dermal layer experienced a progressive process of histological maturation and differentiation in culture [20]. The fact that the dermis was likely capable of protection from UV light is very relevant, especially for UV-A, as this radiation with a longer wavelength can penetrate deeply in the skin and reach the dermal layer, whereas UV-B are mostly absorbed by the most superficial structures and UV-C is quenched by the atmosphere [1]. In this regard, we first found that the bioartificial skin tended to synthesize increasing amounts of proteoglycans, which may be explained by the increasing number of fibroblasts found at the dermal layer. The fact that the artificial skin shows more proteoglycans than CTR skin could be associated with the nature of the biomaterial used in the UGRSKIN model, which contains a natural polysaccharide such as the agarose, which could contain proteoglycans. Our results then showed that the presence of collagen fibers tended to increase with time, although levels were always lower than CTR skin, as previously reported for bioartificial skin kept ex vivo [22,23]. Although the maturation level of the dermal layer was lower than the epidermis, it is probable that the sequential synthesis of dermal fibrillar and non-fibrillar components of the dermis may also play a role in absorbing incoming UV light in bioengineered skin. As the sequential maturation of the epidermis correlated with dermal development, we could state that an orchestrated process of differentiation may be simultaneously occurring in both layers as a result of a dermo-epidermal interaction, as it is the case with the developing skin [46].

## 5. Conclusions

In summary, the present work allowed us to characterize the UGRSKIN model of bioengineered human skin regarding UV light behavior. On the one hand, we found that the epidermal layer was able to mature and differentiate in a time-dependent manner. The artificial skin developed a thick epithelium with several cell strata expressing relevant epithelial proteins, and this epithelium was able to absorb most of the incoming UV light. On the other hand, the fact that the dermis also showed sequential signs of maturation and differentiation suggests that this layer could also play a role in UV light protection. These findings support the use of samples corresponding to 21 or 28 days of development for clinical purposes not only due to their higher structural similarities with native skin, but also because of their optical properties in the UV range. Future studies should determine the biological behavior of the skin substitutes subjected to continuous UV irradiation to ascertain how these bioartificial tissues respond to the incoming light in a setting able to reproduce the in vivo situation.

This study could contribute to the preclinical characterization of the UGRSKIN model, as a thorough characterization is a key requirement of bioartificial tissues intended for clinical use. The demonstration that the bioartificial skin could protect the patient’s inner tissues from the incoming UV light supports the clinical use of the UGRSKIN model. The suitability of this skin model should be compared with other types of skin substitutes generated by 3D printing or other biofabrication methods [47,48].

## Data Availability

All relevant data are shown in the manuscript. Additional data are available on request from the corresponding author.
